# An ecological and theoretical deconstruction of a school-based obesity prevention program in Mexico

**DOI:** 10.1186/s12966-014-0103-2

**Published:** 2014-08-10

**Authors:** Margarita Safdie, Margaret Cargo, Lucie Richard, Lucie Lévesque

**Affiliations:** Centro de Investigación en Nutrición y Salud, Instituto Nacional de Salud Pública, Av. Universidad 655, Sta. Ma. Ahuacatitlán, Cuernavaca, Morelos 62508 México; School of Kinesiology and Health Studies, Queen’s University, 28 Division Street, Kingston, Ontario Canada; School of Population Health, University of South Australia, 160 Currie Street, GPO Box 2471, Adelaide, South Australia 5000 Australia; Faculty of Nursing and IRSPUM, Université de Montréal, Station Centre-ville, PO Box 6128, H3C3J7 Montréal, Quebec Canada

**Keywords:** Children, Physical activity, Nutrition policy, Social cognitive theory

## Abstract

**Background:**

Ecological intervention programs are recommended to prevent overweight and obesity in children. The National Institute of Public Health (INSP) in Mexico implemented a successful ecological intervention program to promote healthy lifestyle behaviors in school age children. This study assessed the integration of ecological principles and Social Cognitive Theory (SCT) constructs in this effective school-based obesity prevention program implemented in 15 elementary schools in Mexico City.

**Methods:**

Two coders applied the Intervention Analysis Procedure (IAP) to “map” the program’s integration of ecological principles. A checklist gauged the use of SCT theory in program activities.

**Results:**

Thirty-two distinct intervention strategies were implemented in one setting (i.e., school) to engage four different target-groups (students, parents, school representatives, government) across two domains (Nutrition and Physical Activity). Overall, 47.5% of the strategies targeted the school infrastructure and/or personnel; 37.5% of strategies targeted a key political actor, the Public Education Secretariat while fewer strategies targeted parents (12.5%) and children (3%). More strategies were implemented in the Nutrition domain (69%) than Physical Activity (31%). The most frequently used SCT construct within both intervention domains was Reciprocal Determinism (e.g., where changes to the environment influence changes in behavior and these behavioral changes influence further changes to the environment); no significant differences were observed in the use of SCT constructs across domains.

**Conclusions:**

Findings provide insight into a promising combination of strategies and theoretical constructs that can be used to implement a school-based obesity prevention program. Strategies emphasized school-level infrastructure/personnel change and strong political engagement and were most commonly underpinned by Reciprocal Determinism for both Nutrition and Physical Activity.

## Introduction

The rate of overweight and obesity in Mexican children is growing at an alarming pace. National Surveys reveal that the prevalence of overweight and obesity in school age children increased from 18.4% in 1999 [1] to 26.2% in 2006 [2] to 30.3% in 2008 [3]. In 2006–2008, the school environment in Mexico was considered to be “obesogenic” because of restricted opportunities for physical activity (PA) and enhanced opportunities to consume energy-dense products [4-6]. High-fat foods and sugar-laden beverages low in nutritional value were available to students during five daily eating opportunities. Given this context, effective strategies to address this serious public health problem are deemed critical.

Ecologically-founded intervention programs that encompass a diversity of strategies to engage different stakeholders in a variety of settings are recommended to prevent overweight and obesity in children [7-9]. In 2006, the National Institute of Public health (INSP) designed and implemented an innovative and successful intervention program to modify the school environment to promote healthy lifestyle behaviors in 4th, 5th, and 6th grade students [10]. This intervention program was designed according to ecological principles that recognize the reciprocal relationship between individuals and their environment [11], based on formative research [4-6], and informed by Social Cognitive Theory [12].

The premise underlying ecological programming is that a multilevel program is likely to be more effective than an individually focused program because it affords the opportunity to encounter the same behavioral prompts (e.g., to be more physically active) from a variety of sources (parents, teachers, coaches) in a variety of settings (home, school, community) [13-16]. Therefore, an intervention program that contains diverse strategies to engage several different stakeholders across a range of settings might address the health behavior in a more comprehensive way and thus yield better results than a simpler program (i.e., fewer targets, less settings). Despite its intuitive appeal and an increase in the use of ecological principles for programming to prevent childhood obesity in developed countries [17], optimal (i.e., effective, easy to implement at low cost) combinations of intervention activities to promote healthy lifestyles have yet to be identified. In addition to determining optimal combinations of intervention activities, health promotion practitioners striving to integrate ecological principles into their programs must also strive to develop programs that are theoretically informed. Challenges to theoretical integration include practitioner difficulties in operationalizing and assessing theoretical constructs [18]. The purpose of this study was to assess the integration of ecological principles and theoretical constructs in a school-based obesity prevention program that was successful in creating a supportive environment for healthy behaviors.

## Background

Conceptual frameworks of the ecological approach present health as resulting from the interdependence between the individual and his or her ecosystems of family, community, physical, social and political environments [19,20]. Health promotion practitioners endeavoring to apply an ecological approach are tasked with considering and leveraging the multiple influences within these ecosystems to guide comprehensive intervention strategies to impact behavior and health. This approach is widely used and accepted for guiding interventions [20,21].

A recent review of the usefulness of an ecological approach shows that most of its applications have been to enhance PA and healthy eating [17]. In the literature, there are examples of effective nutrition and PA initiatives in schools to improve children’s opportunities for health based on an ecological approach [22,23]. However, there is a lack of guidance about combinations of intervention activities to replicate successful intervention efforts, especially in the obesity prevention area.

Social Cognitive Theory (SCT) is widely used for the design of ecologically framed healthy eating and PA intervention programs [24-26]. SCT is consistent with an ecological approach because it postulates a reciprocal relationship between people and their environment; each interact and influence each other [12]. According to Bandura [12], Reciprocal Determinism, a core construct of SCT, is characterized by a series of ecological transactions (i.e., activities, relationships, influences, etc.) between individual level factors (e.g., thoughts, beliefs, and attitudes) and environmental factors (e.g., availability and accessibility to resources) [12]. These transactions yield changes in both the individual and the environment. For example, health promotion staff educate school food vendors to offer healthy foods during recess (environmental change), children purchase and consume the healthy food (individual change), the increase in sales due to the availability of healthy food at recess leads food vendors to add more healthy food options, leading children to purchase more healthy food, and so on.

Another important SCT construct is Self-efficacy (SE), which is defined as the belief a person holds regarding their ability to successfully perform a specific behavior under specific conditions [12]. Another important SCT construct is Behavioral Capacity (BC) to engage in a change, which depends on knowledge (about what do to) and skills (about how to do it) related to a behavior; behavioral capacity is a pre-requisite for self-efficacy and self-confidence. Children with strong self-efficacy and strong behavioral capacity to engage in schoolyard active games are more likely to participate in these activities. Reinforcement (R), another SCT construct, refers to ways in which a preferred behavior can be encouraged or an undesirable behavior can be discouraged. For example, offering free fruit to children at recess, while requiring payment for less healthy options can provide an incentive for making the healthy choice while providing a disincentive to make an unhealthy choice [27]. Even though these constructs have been widely used to describe interactions between behavior, cognition, and the environment there is little guidance to describe how these constructs may be best aligned to achieve behavioral change [18,28-30].

A recently published impact evaluation of a school-based ecological intervention program in Mexico documented improvements in children’s behaviors and the school environment. A detailed description of intervention impacts during its two year implementation is provided elsewhere [10,31,32]. Briefly, 27 schools were randomly assigned to the basic intervention, plus intervention or control condition. The evaluation assessed whether there were differences between intervention and control conditions in the school environment (i.e., food and beverage availability and PA opportunities), children’s health behaviors (i.e., food consumption and steps taken), and children’s body composition. Measures were assessed four times over the two-year period in a sample of 830 students. The availability of healthy foods and beverages increased significantly in the intervention school conditions with a concomitant decrease in unhealthy food availability. In addition, children’s food intake improved and the number of steps taken during the school day increased significantly; body mass index in children and obesity prevalence did not change. Providing a detailed evaluation of the ecological and theoretical elements implemented within this program constitutes a first step in understanding how these behavior and environmental changes occurred. Few evaluations of complex obesity prevention interventions report on the nature of the intervention exposure. A mapping of the integration of ecological principles and theoretical constructs involved in this successful Mexican school-based intervention program can help health promoters in emerging countries like Mexico replicate successful intervention programs in an effective and efficient manner.

Study aims were: 1) to identify the number and type of strategies implemented to encourage student healthy eating and PA at school (e.g., how many strategies aimed to change the food environment? how many strategies aimed to change the PA environment? what were the social and environmental targets for change and how were these configured to eventually impact the children? and, 2) to describe the use of SCT theoretical constructs within the two intervention domains (e.g., were some SCT constructs used more than others? were SCT constructs used differently within the Nutrition and PA domains?).

## Methods

### Context of the study

The INSP school-based obesity prevention program was designed according to a simplified ecological model (SEM) proposed by McLeroy [11], informed by a formative evaluation [4,5 and based on Social Cognitive Theory [12]. Four levels of influence were targeted for change: individual (students), interpersonal (teachers, school staff, and parents), organizational (schools), and political (Secretaria de Educacion Publica, i.e., Public Education Secretariat).

The program included two intervention domains: Nutrition and Physical Activity. Interventions were also supported by a communication and education strategy. A detailed overview of the intervention activities is published elsewhere [10]. In brief, the intervention program was implemented and evaluated using an experimental design in 27 schools (16 intervention, 11 comparison schools) in Mexico City. The intervention program was implemented by INSP staff over approximately 8 months per year for two years during 2007 and 2008. Comparison schools did not receive the intervention program.

The overall purpose of the intervention program was to improve the food and PA school environment as a way to promote healthy behaviors in children. Intervention activities within the Nutrition domain aimed to enhance the availability of healthy food and water at school whereas PA intervention activities aimed to increase opportunities for moderate-to-vigorous PA (MVPA) during recess and Physical Education (PE) classes at school. The aim of the communication and education strategy was to support healthy practices at schools; to promote positive attitudes, and values towards healthy behaviors among teachers, food vendors, parents, and children through educational activities and persuasive messaging. Examples of intervention activities included providing lists of authorized foods that could be sold by food vendors during recess, providing morning fitness activities to the entire school before morning announcements, providing activity kits of sports equipment to be used during recess, teacher training workshops, and posting information in classrooms about healthy eating and PA (see Table 1 “INSP” labelled activities for additional examples).

**Table 1:** **Target types, intervention strategies and related intervention examples**

**Target type**	**Intervention strategy**	**Description** **/** **example**
INDIVIDUAL	HP → IND	Interventions aimed at changing children directly
		E.g., Offering workshops for students on how to eat healthy^INSP^
	HP → (IND—IND)	Interventions aimed at linking up children to perform activities together
		E.g., Finding a partner for sports at recess*
INTERPERSONAL	HP → INT → IND	Interventions aimed at changing children’s interpersonal environment (people who can influence the IND, e.g., friends, parents, siblings)
		E.g., Distributing booklets to parents on how to pack a healthy lunch^INSP^
	HP → (INT—INT) → IND	Interventions aimed at linking up families/small groups to actually participate in activities together
		E.g., Healthy cooking group that meets to learn and cook healthy recipes*
ORGANIZATIONAL	HP → ORG → IND	Interventions aimed at changing children’s organizational environment (school infrastructure and/or school personnel)
		E.g. Improving school premises to promote PA^INSP^
	HP → (ORG—ORG) → IND	Interventions aimed at linking organizations together
		E.g., Creating a partnership between two schools to buy sports equipment*
COMMUNITY	HP → COM → IND	Interventions aimed at changing the community environment (community infrastructure and/or community members)
		E.g. Improving parks to engage in PA after school*
	HP → (COM—COM) → IND	Interventions aimed at linking communities together
		E.g., Developing a partnership of two neighborhoods to clean the park to engage in PA*
POLICY	HP → POL → IND	Interventions aimed at influencing political representatives to legislate for the promotion of physical activity
		E.g. Lobbying activities for the development of policies for safer active transportation to school*
	HP → (POL—POL) →IND	Interventions aimed at creating alliances to promote more effective intergovernmental cooperation
		E.g., Brokering an alliance between federal and local authorities for the delivery of PE classes in schools*
MIXED	HP → POL → ORG → IND	Interventions aimed at changing children through multiple targets
		E.g. Creating a policy mandating all teachers to attend training to deliver physical activation sessions to students^INSP^

*HP* = Health Promotion Delivery Agent, *IND* = Individuals, *INT* = Interpersonal environment, *ORG* = Organizational environment, *COM* = Community environment, *POL* = Political environment, → = Direction of intervention activity transfer between the change agent and its intended target.

^INSP^Examples drawn from the National Institute of Public Health (INSP) school-based obesity prevention program; *Examples drawn from [13].

Trans-Domain policy activities included meetings with the Public Education Secretariat at the provincial and federal levels to improve the practices and guidelines regarding PE classes and healthy eating in schools. Organizational activities included improving the quality of food and beverages available at schools through a recommendation list and food vendor training; improving the school infrastructure to be more PA friendly; and, posting and distributing communication materials at schools. Interpersonal activities included meetings with parents and the distribution of printed educational materials. Individual activities included dietary and PA workshops targeted to children.

### Data collection

For the purposes of this analysis, we examined the intervention activities implemented across 15 intervention schools in year 2 of the program (due to a program change, one of the intervention schools no longer met the inclusion criteria in year 2). We focused the analysis on year 2 activities because the intervention activities had been refined from year one, intervention staff had had more experience, and adherence to the program model was enhanced relative to year one. Relationships between the Public Education Secretariat, school staff, and INSP were also better established during the second year.

Data collection comprised two components. One component resulted in the compilation of relevant information about the implementation of intervention activities in schools while the second data collection component consisted of determining the use of the SCT constructs by project staff. All project documents (i.e., project intervention and Public Education Secretariat meeting minutes, implementation journals, monitoring forms, institutional communications between the Public Education Secretariat, participating schools, and the INSP) related to the Nutrition and PA domains, were compiled and sorted into standardized reports describing the intervention in a detailed manner according to setting, target group and objectives of the intervention. This yielded 25 distinct activity implementation reports across the intervention schools. These reports were compiled by the main researcher/project coordinator of the program and two supervisors. To complement missing information and to validate the content of activity implementation descriptions, we conducted discussion groups with 15 project staff who were in charge of specific strategy execution or who played key roles during the implementation (e.g., supervisors). In addition, implementers were asked to complete a 25-item checklist [33] to report on the SCT constructs addressed in the implementation of intervention activities within each intervention domain (Nutrition and PA). This study was cleared by the General Research Ethics Board at Queen’s University, Kingston, Ontario, Canada.

### Measures

The Intervention Analysis Procedure [34] was used to assess the integration of ecological principles in the intervention program and to create a program “map”. The Intervention Analysis Procedure is based on Richard and colleagues’ scheme to identify *intervention settings*, *targets*, and *strategies* as three key dimensions through which greater integration of an ecological approach can be operationalized in health promotion programming [15]. Inspired from Miller [35], Richard and colleague’s scheme recognizes four types of intervention settings: organizational (ORG), defined as entities characterized by a formal hierarchy (e.g., schools, business); community (COM), defined as a group of persons and/or organizations in a specific area (e.g., neighborhoods, parent associations); society (SOC) defined as a larger system that has control over those located in their constituencies (e.g., states); and supranational (SUPRA) defined as two or more societies (e.g., the European Union). The health promotion (HP) delivery agent may intervene in one or more of these settings.

Building on McLeroy’s [11] work, Richard et al’s scheme defines intervention *targets* as the intended beneficiaries of the HP intervention activities and identifies five possible targets: individuals (IND, e.g., children); the interpersonal environment (INT, e.g., people from one’s personal network who have influence over the IND); the organizational environment (ORG, e.g., school infrastructure and/or school personnel); the community environment (COM, e.g., community infrastructure and/or community members); and, the political environment (POL, e.g., policies or elected representatives). The “ultimate target” of an intervention is always individuals (IND) who can be engaged *proximally* by the HP (e.g., HP provides health information to the IND), or *intermediately* via other targets [(e.g., HP provides training to teachers (ORG) so that they can encourage healthy behaviors in children (IND)].

The intervention *strategy* represents the relationship that joins the targets intended for change with the intervention program [15,36]. Multiple targets within a given strategy can be joined by either a direct transformation relationship, or by a networking relationship. A direct transformation relationship is denoted graphically by an arrow linking the HP to the ultimate target (e.g., HP → IND) or to its intermediaries (e.g., HP→ORG→IND). A networking relationship is when the HP brokers a new relationship between two or more entities in order to influence the ultimate target; it is graphically depicted by brackets surrounding the entities in the network (e.g., HP→ [ORG-ORG] →IND). Network relationships can take the shape of different collaborative partnerships such as interagency alliances, community coalitions, informal cooperatives, and advocacy groups [15]. These two types of relationships can be used in diverse combinations and might involve numerous targets before reaching the ultimate target. Table 1 shows examples of some of these intervention strategies by target; examples are drawn from the INSP program and complemented by examples from Gauvin and colleagues [14].

We used the Intervention Analysis Procedure [34] to identify the intervention setting, targets, and strategies contained within the activity descriptions. Two coders were trained by Richard in the application of the Intervention Analysis Procedure and independently coded the 25 activity intervention reports with an initial concordance of 93%. Coding discrepancies were related to different interpretations of intervention targets and were resolved by consultation with the co-authors (“experts”).

The theoretical construct checklist [33] completed by project staff was used to assess the integration (i.e., presence/absence) of SCT theoretical constructs within the implementation of activities for each intervention domain (i.e., Nutrition and PA). The 25 item-checklist comprised four SCT constructs, including six Self-Efficacy items (e.g., did activities integrate verbal motivation?); eight Reciprocal Determinism items (e.g., did activities aim to change existing practices or existing messages?); five Behavioral Capacity items (e.g., did activities aim to correct misconceptions?); and, six reinforcement items (e.g., did activities include incentives to participate?). Construct-specific items were grouped into four indices, and the within-index total was divided by the total number of index-specific items, yielding a construct-specific score.

### Data analysis

Given the low frequency of some strategies within each domain (i.e., < 5), only descriptive statistics (percentages) were used to examine distinct intervention strategies and targets by domain. We used non-parametric analyses to describe the integration of SCT theoretical constructs between and within Nutrition and PA intervention domains. To determine differences in SCT construct use *between* domains, we used the Wilcoxon ranked test and to identify differences in SCT construct use *within* domains, we used the Friedman test (SPSS version 19 IBM, Chicago).

## Results

### Ecological programming

The INSP school-based obesity prevention program implemented 32 distinct intervention strategies in schools during the 2007–2008 school year. All of the intervention strategies (i.e., 100%) occurred within a school setting (ORG) where the ultimate target (i.e., children) was reached.

The intervention program targeted the Public Education Secretariat (POL), schools/teachers (ORG), parents (INT), and children (IND). The intervention program only used direct transformation relationships, either by targeting the children proximally or by transforming the environment or actions of intermediaries of the children. Descriptive results displayed in Table 2, show that overall, four different types of strategies were used, where 12.5% of the strategies targeted children (IND); 3% of the strategies targeted parents (INT); 47% of the intervention strategies targeted the school infrastructure and/or personnel (ORG); and 37.5% of the interventions targeted the Public Education Secretariat (POL) with the aim of modifying the school infrastructure and/or personnel (ORG). Moreover, our results show that 69% of the intervention strategies were implemented within the Nutrition domain, whereas 31% of strategies were implemented within the PA domain.

**Table 2:** **Intervention strategy by intervention domain**

**Intervention domain**			
	**Nutrition**	**Physical activity**	**Total**
Intervention strategy			
HP →IND	3	1	4
HP → INT → IND	1	0	1
HP → ORG → IND	12	3	15
HP → POL → ORG → IND	6	6	12
Total	22	10	32

HP → IND: Health Promotion (*HP*) delivery agent intends to promote change in Individuals (IND).

HP → INT → IND: Health Promotion (*HP*) delivery agent intends to promote change in the Interpersonal environment (INT) in order to promote change in Individuals (IND).

HP → ORG → IND: Health Promotion (*HP*) delivery agent intends to promote change in the Organizational environment (ORG) in order to promote change in Individuals (IND).

HP → POL → ORG → IND: Health Promotion (*HP*) delivery agent intends to promote change in the Political environment (POL), in order to promote change in the Organizational environment (*ORG*), in order to promote change in Individuals (IND).

### Use of Social Cognitive Theory constructs

**Figure 1:**
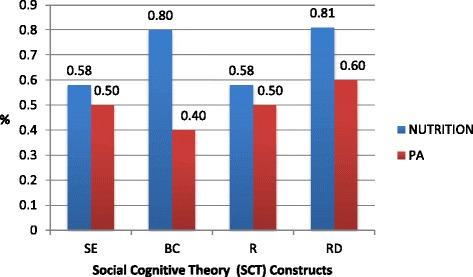
**Social Cognitive Theory construct use within the Nutrition and Physical Activity (PA) intervention domains.**

Overall, results presented in Figure 1 show that the construct used most frequently within both intervention domains was Reciprocal Determinism (RD). However, within the Nutrition domain, Behavioral Capability (BC) was used to the same degree as RD, while Self-Efficacy (SE), and Reinforcement (R) were used less frequently, but equally. The pattern of construct use was different for the PA domain; in general, all of the constructs were used less often than they were within the Nutrition domain. However, none of the constructs in particular appear to have been favored; each being used approximately with the same frequency. The Wilcoxon test revealed no significant differences in SCT constructs (SE Z = −.586, p = .558; RD Z = −1.41, p = .254; R Z = −1.611, p = .107; BC Z = 1.81, p = 0.70) use between the two domains and the Friedman test revealed no significant differences in the construct use within Nutrition (*X*^*2*^ = 6.35, p = 0.096) and PA (*X*^*2*^ = 6.94, p =0.074) domains.

## Discussion

The aim of this paper was to assess the integration of ecological principles and theoretical constructs involved in a successful school-based obesity prevention program in Mexico City. We sought to unpack the intervention program along intervention domains in order to develop a map of the successful intervention program. If we compare our ecological map with recommendations in the literature [15,17,20], we can ascertain that this intervention program was a genuine ecological effort within a single setting since it delivered a diversity of intervention strategies involving multiple targets (POL, ORG, INT, IND), across both intervention domains. The ecological mapping showed that several different targets (PE teachers, school teachers, food vendors, parents, children and the school environment) were engaged, and that these efforts are consistent with those recommended to promote behavior change and to prevent obesity in children [7,9,23,37-39].

Our findings indicate that the project was only implemented within the school (ORG) setting; this finding is not surprising given that this was a school-based intervention program. It appears that the intensity of effort invested in the implementation of intervention activities across the two domains, varied. Within the Nutrition domain, more intervention strategies were delivered overall, relative to the PA domain. This finding is not unexpected given that when the project was implemented, the nutrition and PA contexts in the schools were different [9,10]. The nutrition environment was disadvantaged in relation to the PA environment; for instance, there were no nutritional guidelines to regulate eating practices at school, whereas PE class, even though only offered once a week, was already in place as a regular, mandated practice [40]. Thus, there may have been more “room for improvement” for INSP staff to influence nutrition in schools. We found that more than half of the strategies within the Nutrition domain were focused on organizational change, while the rest were mainly aimed at influencing policy to impact schools. Given significant improvements in food intake in children and food availability at schools [31], it would appear that the use of a diversity of strategies at different ecological levels was effective.

Overall, we observed that in relation to the intervention strategies implemented within the Nutrition domain, strategies implemented within the PA domain were less in number and in diversity, with the majority of strategies concentrated on targeting the Public Education Secretariat (POL) as a way to improve PE and PA at schools. This finding is not surprising given that the improvement of PE and PA opportunities typically requires an injection of new resources (e.g., new equipment, additional PE teachers) [41], whereas changes to the food environment do not necessarily require *more* resources per se, but instead, require an improvement of practices (e.g., replacing energy-dense foods with fresh fruits and vegetables [10]). Furthermore, within the Mexican context, curriculum change is not within the mandate of schools, but rather belongs to the state. Thus, the main way in which PE can be impacted in a sustainable manner at schools is through policy change at the state level (Public Education Secretariat). Given that during the 2007–2008 year period, the proportion of students taking more steps (according to pedometer counts) increased in the intervention schools as compared to comparison schools (p = .06) [31], it would seem that strategies to engage policy decision-makers to influence school PA opportunities may be particularly warranted in Mexico.

Overall, our findings are consistent with existing evidence that policy intervention strategies can impact different ecological levels of influence [42]. This may be especially the case for hierarchical institutions such as school systems, where decisions are made at more than one level.

Finally, another notable finding from the current study is that only one intervention activity was implemented to engage parents in the support of healthy lifestyles for their children. Given growing evidence that family support is essential to the success of school-based health promotion activities [41,43], the lack of family oriented intervention activities would appear to be a shortcoming of the INSP program. Based on the existing literature [44-46], it can be speculated that the inclusion of additional intervention activities to engage families in obesity prevention efforts might have resulted in a larger impact in child PA and nutrition behavior change. Future research should investigate the feasibility of engaging families to support school-based health promotion efforts in Mexico.

An analysis of SCT behavioral constructs showed no statistical differences in their use within and between intervention domains. However, some tendencies and variations were observed. The theoretical construct most often used by intervention staff to influence the different ecological levels was reciprocal determinism (RD), also referred to as the environmental construct [12,22,23,30,38]. Given that the aim of the intervention program was to improve the environment, the frequent use of this construct is not surprising. Intervention activities flowing from the RD construct intended to influence existing attitudes about the school food and PA environment, related policies, practices, and services. For instance, HP workers supported the improvement of the food environment by influencing the reduction of the sale of sweets during recess and by facilitating conditions for water consumption by organizing potable water deliveries to schools. HP workers improved the PA environment and modeled schoolyard use by organizing structured games during recess. The use of the RD construct to influence the food and PA environment has been shown to be effective for creating a supportive milieu for healthy behaviors in children [47].

Patterns of use of other SCT constructs appeared to vary slightly, although not significantly, across and within domains. Within the Nutrition domain, BC was used as frequently as RD. In the Nutrition domain, the BC construct was used in workshops to influence food and nutrition knowledge and to model skills that children need to carry out healthy eating behaviors. In addition to providing verbal prompts to intervention targets, HP workers used BC-oriented print materials to engage food vendors, key authorities and children as a way to correct misconceptions about healthy eating and to provide new options for improving food related practices at school. The frequent use of RD within the Nutrition domain was likely related to program aims to influence food availability at schools. The high use of these two constructs in the Nutrition domain is congruent with the INSP impact results that show significant improvements in the food environment and enhanced healthy food intake by children in intervention schools [31]. These results are consistent with other studies that have effectively used these constructs to improve food intake practices in children [47-49].

Within the PA domain, the use of SCT constructs appeared to be balanced. This may be because PE sessions were already occurring in schools on a regular basis and were being delivered by professionally trained experts (i.e., PE specialists). Thus, the implementation of activities within the PA domain may have required less guidance by INSP staff as compared to activities within the Nutrition domain, which required that food vendors be provided with guidance and information to learn new practices about how to create a healthy food environment. This finding is comparable to other research showing less use of theoretical constructs in the implementation of PA in interventions for children relative to other domains and other populations [50].

Overall, SCT behavioral constructs were used less frequently in the PA domain relative to the Nutrition domain, although this difference was not significant. This finding is consistent with our ecological mapping showing that the majority of PA intervention strategies were targeted to the Public Education Secretariat (POL) and aimed to modify the political environment rather than to change the behaviors of individuals (e.g., students and school staff). It may be that constructs from theories other than SCT (e.g., relevant to the policy process) are used to influence policy.

Our findings provide new insight into a promising combination of strategies and theoretical constructs that can be used to implement an ecologically founded school-based obesity prevention program. A limitation of this study was our inability to gauge the magnitude of effort devoted to various intervention activities. The IAP does not allow for the assessment of the “dose” of each intervention. Thus, the development and implementation of educational materials to enhance healthy eating might have been captured as one intervention activity (and coded as a single strategy), when in fact these same materials might have been used daily by teachers. In addition, the checklist used to assess SCT construct use only captured four of the SCT constructs; a more exhaustive list may have yielded a different theoretical picture.

## Conclusions

To our knowledge, this is the first research to document the integration of ecological principles and theoretical constructs in a school-based obesity prevention program in Mexico. The deconstruction of a successful intervention program that has documented environmental and student behavior improvements provides novel information for the implementation of multifactorial interventions in school-based health promotion programs. Although there may be a variety of successful combinations of ecological strategies and theoretical constructs, our findings provide one version that can be used as a starting point to develop even more effective combinations. Within the school setting, this ecological combination of strategies emphasizes school and political targets. Moreover, the strategies in both the Nutrition and Physical Activity domains are most commonly underpinned by the theoretical construct of Reciprocal Determinism. In a context where the school environment is considered “obesogenic” and there is compelling evidence that this environment can shape children's eating and PA patterns, the current findings provide valuable insight about the types of strategies that can be leveraged to optimal effects. It is expected that these findings will be especially meaningful to inform obesity prevention programs in Mexico and in low-middle income countries where childhood obesity is an emerging problem.

## Abbreviations

BC: Behavioural capability
COM: Community
HP: Health promotion
IAP: Intervention analysis procedure
IND: Individual
INSP: National Institute of Public Health
INT: Interpersonal
MVPA: Moderate to vigorous physical activity
ORG: Organization
PA: Physical activity
PE: Physical education
POL: Political
R: Reinforcement
RD: Reciprocal determinism
SA: Secretary of Health
SCT: Social cognitive theory
SEM: Socioecological approach
SUPRA: Supranational
